# The interrelated roles of RAB family proteins in the advancement of neoplastic growth

**DOI:** 10.3389/fonc.2025.1513360

**Published:** 2025-03-24

**Authors:** Yuxin Ji, Ruonan Li, Guohui Tang, Wenrui Wang, Changjie Chen, Qingling Yang

**Affiliations:** ^1^ Anhui Provincial Key Laboratory of Tumor Evolution and Intelligent Diagnosis and Treatment, Bengbu Medical University, Bengbu, Anhui, China; ^2^ Clinical Testing and Diagnose Experimental Center, Bengbu Medical University, Bengbu, Anhui, China; ^3^ Department of Biotechnology, Bengbu Medical College, Bengbu, Anhui, China; ^4^ Institute of Health and Medicine, Hefei Comprehensive National Science Center, Hefei, Anhui, China

**Keywords:** RAB proteins, cancer progression, vesicular trafficking, glucose metabolism, autophagy, drug resistance

## Abstract

Rab Proteins, A Subfamily Of The Ras Superfamily Of Small Gtpases, Are Critical Regulators Of Intracellular Vesicular Trafficking, Which Is Intricately Linked To Various Cellular Processes. These Proteins Play Essential Roles Not Only In Maintaining Cellular Homeostasis But Also In Mediating The Complex Interplay Between Cancer Cells and Their Microenvironment. Rab Proteins Can Act As Either Oncogenic Factors Or Tumor Suppressors, With Their Functions Highly Dependent On The Cellular Context. Mechanistic Studies Have Revealed That Rab Proteins Are Involved In A Variety Of Processes, Including Vesicular Transport, Tumor Microenvironment Regulation, Autophagy, Drug Resistance, and Metabolic Regulation, and Play Either A Promotional Or Inhibitory Role In Cancer Development. Consequently, Targeting Rab Gtpases To Restore Dysregulated Vesicular Transport Systems May Offer A Promising Therapeutic Strategy To Inhibit Cancer Progression. However, It Is Equally Important To Consider The Potential Risks Of Disrupting Rab Functions, As Their Roles Are Highly Context-Dependent and May Have Opposing Effects In Different Malignancies. This Review Focuses On The Multifaceted Involvement Of Rab Family Proteins In Cancer Progression Underscores Their Importance As Potential Therapeutic Targets and Underscores The Need For A Deeper Understanding Of Their Complex Roles In Tumorigenesis.

## 1 Introduction

RAB GTPases, the largest branch of the Ras protein superfamily, have regulatory factor functions in vesicular transport, endocytosis membrane transport, membrane targeting and fusion. In humans, more than 70 different RAB proteins are localized in different intracellular membranes (1). As master regulators of intracellular cargo transport, RAB proteins precisely control vesicle formation, movement, tethering, and fusion, thereby maintaining cellular homeostasis and signal transduction. In recent years, growing evidence has demonstrated that RAB proteins are not only essential for normal cellular physiology but also play complex and multifaceted roles in tumor initiation, progression, and metastasis.

Tumor development is a multifactorial and multistep process involving the dysregulation of various biological behaviors, including cell proliferation, apoptosis, metabolism, invasion, and migration. Studies have shown that the dysregulation in RABs level or the interaction between RABs and effectors may be associated with cancer (2). In some scenarios, RAB proteins may act as oncogenic factors, promoting tumor progression by enhancing invasiveness, metastatic potential, or drug resistance; in other contexts, they may function as tumor suppressors, limiting tumor growth by inhibiting proliferation or inducing apoptosis (3). RAB proteins influence tumor progression directly or indirectly by regulating vesicular transport, autophagy, metabolic reprogramming, and interactions with the tumor microenvironment (4).

This review aims to systematically summarize the dual roles of RAB proteins in tumor progression, with a focus on their molecular mechanisms in vesicular transport, tumor microenvironment modulation, autophagy, metabolic reprogramming, and drug resistance. By delving into the multifaceted functions of RAB proteins in cancer, we hope to provide new insights into their roles in tumorigenesis and progression, as well as a theoretical foundation for developing RAB protein-targeted therapeutic strategies.

## 2 The structure and function of RAB proteins

RAB proteins primarily regulate vesicle budding, transport, tethering, and fusion through the transition between GTP- and GDP-bound states (5). Typically composed of approximately 200 amino acids, they feature highly conserved GTP/GDP-binding sites and GTPase activity domains. Structurally, RAB proteins consist of four conserved guanine nucleotide-binding domains and an effector domain. Evolutionarily, RAB proteins exhibit a high degree of conservation, with sequence homology ranging from 55% to 75% across different species (6).

**Figure 1:**
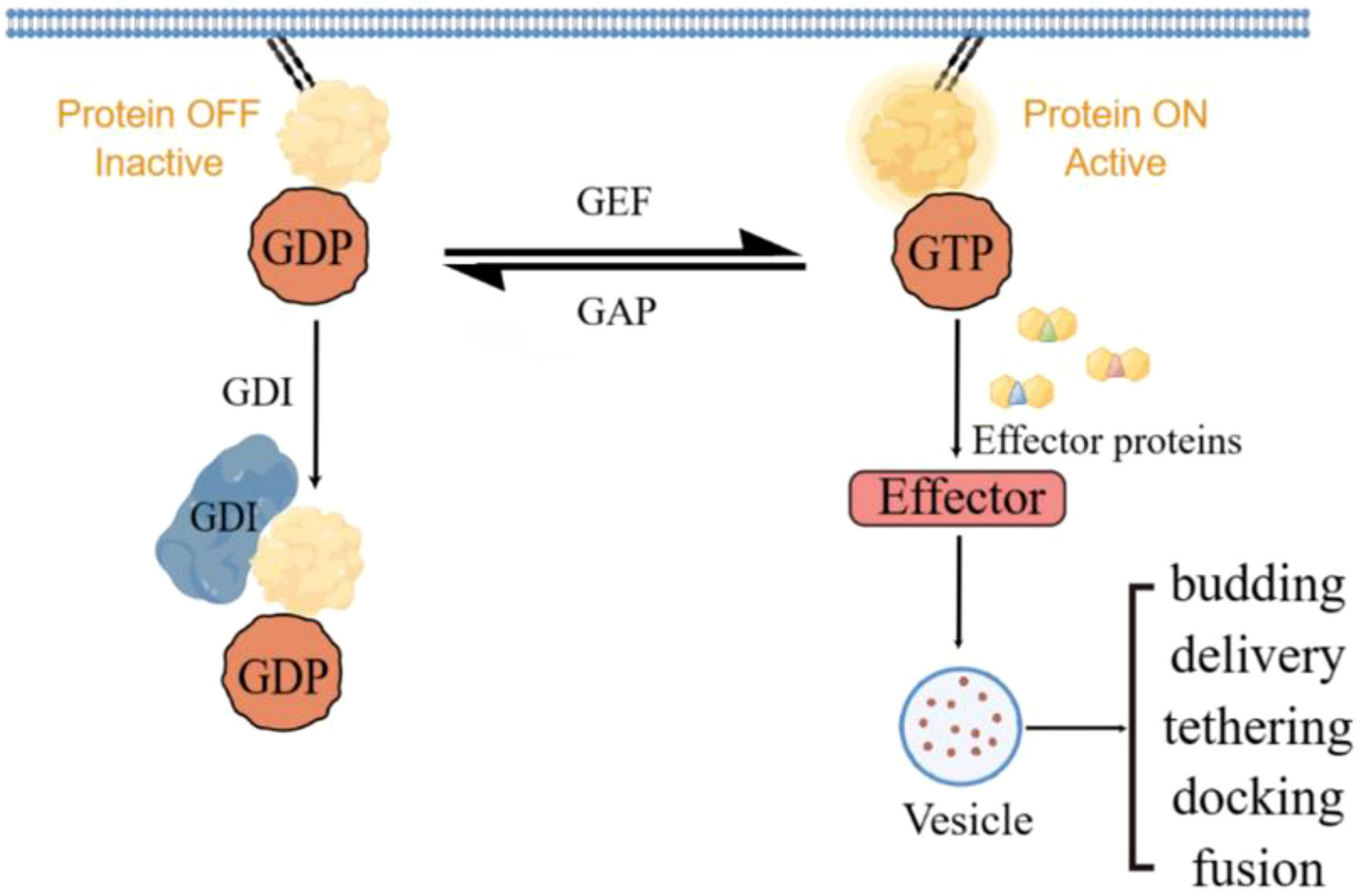
RAB protein cycles between active and inactive and mediates vesicle transport via effector proteins.

RAB GTPases acts as molecular switches to regulate vesicular transport from the cell membrane as well as membrane fusion of target compartments through the regulation of GEFs and GAPs (7). By activating RAB proteins, GEFs may promote membrane transport processes in tumor cells, thereby affecting tumor invasion, metastasis, and drug resistance. GAP may have a dual role by inhibiting the activity of RAB proteins. On the one hand, it may exert an oncogenic effect by inhibiting the activity of tumor-associated RAB proteins; on the other hand, if the function of GAP is inhibited, it may lead to the aberrant activation of RAB proteins, which may promote tumor progression. GDI, by regulating the cycling of RAB proteins, may affect the metabolism and signaling of tumor cells (**Figure 1**).

The critical roles of GAP, GEF and GDI in the regulation of RAB proteins make them potential targets for cancer therapy. By inhibiting the activity of GEF, the activation of RAB proteins can be reduced, thereby inhibiting the membrane transport and invasive ability of tumor cells. The development of drugs that enhance the activity of GAP may help to inhibit the aberrant activation of RAB proteins, thereby exerting an anticancer effect. By regulating the function of GDI, the cycling and distribution of RAB proteins can be influenced, which in turn affects the metabolism and signaling of tumor cells.

## 3 RAB proteins as key regulators of vesicular trafficking

The primary means of regulating the composition and arrangement of the cell surface is through vesicular trafficking, which encompasses endocytosis and endosomal circulation. RAB GTPases are part of a vast family of proteins that are highly conserved and play a crucial role in regulating vesicular trafficking (8).

The RAB GTPases can coordinate various types of responses by activating a variety of effectors at key stages of membrane traffic, such as the budding of vesicles, their delivery, tethering, docking, and their fusion (9, 10). RABs that are GTP-bound can recruit effector proteins to mediate the budding of vesicles in donor organelles, and tether them to acceptor compartments (11). As a result, RAB proteins coordinate spatially and temporally specific vesicle transport, as well as protein secretion and endocytosis. Thus, RAB proteins are known to play a crucial role in intracellular trafficking.

**Figure 2:**
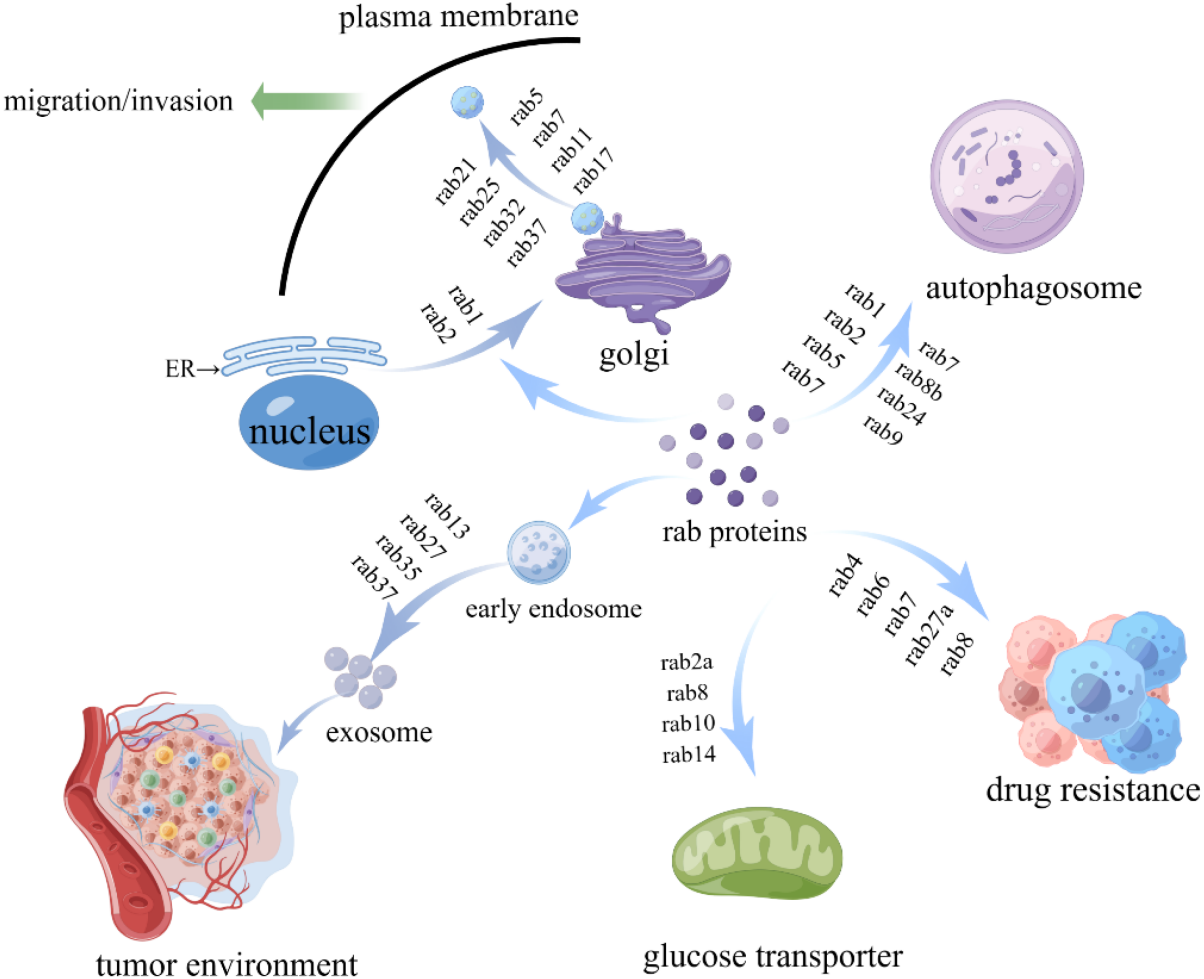
The functions of RAB proteins in tumor development. RAB proteins participate in tumor genesis and development by regulating vesicle trafficking, tumor microenvironment, glucose metabolism, and autophagy.

In tumorigenesis, RAB proteins are functionally impaired by mutations or post-translational modifications that disrupt vesicle trafficking, leading to dysregulated intracellular vesicular transport and contributing to tumorigenesis (2). The relevant RAB proteins are summarized in **Table 1** and **Figure 2**. For example, RAB5 is beneficial to the early stages of endosome formation, and microtubule-dependent adhesion disassembly mediates RAB5 function in vesicle formation and early endosome formation (12). The overexpression of RAB5 promoted the capacity of invasion, migration and exosome secretion in cancer (13). The aberrant expression of RAB5 enhances tumor cell invasion by stimulating endocytosis or cytosolic recycling to promote the release of matrix met alloproteinases(MMP) (14). RAB5 can regulate EGFR vesicular recycling to promote migration in triple-negative breast cancer (TNBC) (15). High RAB2A expression has been found in human breast cancer, and RAB2A regulates most significantly post-endocytosis transport of membrane type 1 (MT1)-MMP and E-calmodulin-polarized Golgi transport by binding to VPS39, suggesting that RAB2A may be an independent predictor of disease recurrence in breast cancer patients (16).

**Table 1:** The functions of RAB proteins in tumor.

Protein	Cancer types	Functions	Ref
RAB5	Lung cancer Breast cancer	① mediate vesicle formation ② promote cell migration and invasion ③ regulate EGFR vesicular recycling to promote migration in TNBC	(3) (3)
RAB7	Prostate cancer Ovarian cancer	① facilitates troglitazone to prevent HGF-induced protease secretion, and prostate tumor growth and invasion ② as regulator of vesicular trafficking ③ promotes cell apoptosis, signaling, migration, and lysosomal degradation in autophagy	(17) (17)
RAB11	Gastric cancer Breast cancer Colorectal cancer	① The increased expression of RAB11 is associated with nodal metastasis in gastric cancer tissues ② the regulator of vesicular trafficking ③ RAB11 transport stimulates breast cancer cell invasion ④ affects the survival, progression and metastatization as well as the accumulation of toxic materials of cancer cells	(12) (12) (80) (22)
RAB15	Neuroblastoma lung cancer	① mediates vesicle trafficking of cell ② impairs cell proliferation, migration and receptor recycling	(81) (82)
RAB17	Liver cancer Endometrial carcinoma kidney cancer	① inhibits the proliferation and migration of hepatocellular carcinoma cells and reduces the tumor growth through ERK signaling pathway ② vesicle docking and fusion ③ correlates with DNA methylation and immune infiltration	(81) (24) (83)
RAB21	Cervical cancer	① affects glucose uptake ② increases autophagic flux	(74) (74)
RAB23	Breast cancer	① localizes to autophagosomes and inhibits cell growth and proliferation and induces apoptosis in breast cancer cells	(59)
RAB25	Ovarian Breast cancer	① medicates integrin to facilitate cancer progression ② promote integrin recycling from late endosomes/lysosomes ③ governs cell-surface receptors recycling and cellular signaling pathways activation	(68) (78)
RAB32	Liver cancer Lung cancer Ovarian cancer Colorectal cancer	① regulate proliferation and metabolism by binding to lysosomes ② regulating phagosome maturation	(68) (84)
RAB37	Lung Cancer Gastric cancer	① Support immunosuppressive TME ② intracellular vesicle trafficking and exocytosis ③ promotes autophagosome formation	(85)
RAB39A	Cervical cancer Colorectal cancer	① regulation of autophagy and lysosomal fusion ② promote cancer stemness and tumorigenesis	(55) (5)
RAB13	Breast cancer Ovarian cancer Colorectal cancer	① regulate the secretion of sEVs classical exosome markers ② GLUT4 vesicle mobilization	(33) (86)
RAB20	Hepatocarcinogenesis Pancreatic cancer Bladder cancer	① regulate the phagosomal acidification ② promotes cell proliferation, migration and colony formation *in vitro* and tumor growth *in vivo*	(87) (88)
RAB35	Hepatocellular carcinoma. Gastric Cancer lung cancer Cervical cancer Breast cancer	① supports tumor microenvironment ② regulates GLUT4 transport ③ enhances the expression of integrin and promotes cell migration	(40) (75) (40)

The RAB7 protein is extensively studied as regulator of vesicular trafficking. RAB7 triggers pro-tumor or anti-tumor effects and exerts oncogenic and oncostatic functions during cancer progression (17). Studies have shown that inhibition of RAB7 expression enhances the pro-apoptotic effects of drugs on TNBC in triple-negative breast cancer, and targeting RAB7 impairs autophagic fluxes to inhibit melanoma growth; thus, targeting RAB7 may contribute to effective cancer therapy (18). RAB7 can facilitate troglitazone to prevent HGF-induced protease secretion, and prostate tumor growth and invasion (19). In the plasma membrane, RAB11 promotes endosome formation and fusion (20, 21). Alterations in RAB11 isoforms significantly affect cancer cell survival, progression and metastasis as well as accumulation of toxic substances (22). In cervical cancer, Rab11 affects tumor cell invasion and migration by regulating vesicle trafficking and the integrin αvβ3/FAK/PI3K pathway signaling pathway (23). As part of the transcytotic machinery,RAB17 docks apically and helps fuse apical vesicles (24). Researchers have found that RAB17 is recruited rapidly to efferosomes, and then migrates to the cell center, promoting cancer proliferation and migration (25). RAB17 can suppresses proliferation and migration of hepatocellular carcinoma cells and reduces the tumor growth via ERK signaling pathway (26). RAB25 can promote or block tumor growth. Integrins can be reinjected from late endosomes/lysosomes through RAB25, which facilitates cancer progression (27). In ovarian cancer, Rab25 enhances cancer cell invasiveness via the expression of the β1 integrin/EGFR/VEGF-A/Snail signaling axis and myostatin (28). In colorectal cancer, Rab25 suppressed the invasion of colon cancer cells by inactivating EGFR through up-regulation of claudin-7 expression (29). Rab34 mediates its vesicular transport within cells by interacting with integrin β3, which in turn regulates tumor cell adhesion and migration (30). RAB37 regulates vesicle trafficking from the Golgi network to the plasma membrane, so it is a regulator of vesicle trafficking and protein transportation (2). There is evidence that RAB37 regulates intracellular vesicle trafficking through GTP and reduces tumor growth/metastases and elicits an immunostimulatory TME (31). RAB39A is a RAB small GTPase located in different subcellular compartments that regulates the endocytic trafficking pathway. Rab39A inhibits cervical cancer cell migration, invasion and epithelial-to-mesenchymal transition EMT by suppressing AKT signaling (32).

The RAB protein plays a crucial role in the regulation of vesicular transport at multiple steps. There is a relationship between tumor progression and deregulation of RAB proteins and destruction of vesicle trafficking networks (5). Targeting RAB proteins can promote or inhibit the proliferation, migration ability of tumor cells by affecting the vesicular transport process. Therefore, the recovery of the dysfunctional vesicular transport system in cancer cells may provide a future direction for inhibiting tumor progression.

## 4 The function of RAB proteins in tumor microenvironment

Extracellular vehicles (EVs) are a heterogeneous group of membrane-structured vesicles that are actively released by almost all types of cells. Small extracellular vesicles (sEVs) usually refers to EVs smaller than 200 nm in diameter and play crucial roles in tumor development, growth, and metastasis by interacting with the tumor microenvironment (33). The tumor microenvironment (TME) consists of many different cells that support or inhibit tumorigenesis and are connected to each other to promote disease progression. The tumor microenvironment can modulate antitumor immunity and contribute to heterogeneity in treatment response. Cancer cells can shape the microenvironment, and stromal components interact with tumor cells to support tumor growth and progression to help support tumorigenesis (34). The morphology and function of cancer cells in the tumor microenvironment are altered, and the diversity of cell types and metabolic patterns may be detrimental to treatment and lead to tumor recurrence.

The RAB proteins are involved in antigen presentation and play a critical role in tumor microenvironment interactions. RAB proteins can foster immunosuppressive TME by regulating the changes in cell interaction factors, thus promoting the progression of cancer. RAB effectors appear to promote tumor development or suppress tumorigenesis through oncogenic signaling pathways. The relevant RAB proteins are summarized in **Table 1**, **Figure 2**. Ectopic RAB5 is differentially expressed in various tumors, and the potential mechanisms include RAB5-regulated exosome secretion from cancer cells and stromal cells, affecting the communication between cancer cells, immune cells and the microenvironment, forming a tumor microenvironment conducive to the growth of tumor cells, and regulating cancer cell migration and invasion (13). RAB6B is a member of the RAB GTPases and has been found to be dysregulated in various tumors. RAB6B expression was found to be associated positively with immune cell infiltration in hepatocellular carcinoma (HCC), while RAB6B expression was associated with CD8+ T cell depletion, leading to an immunosuppressive microenvironment. It suggests that RAB6B may be involved in ECM remodeling in TME, which is associated with the formation of an immunosuppressive microenvironment in HCC. Targeting RAB13 can affect the relationship between cancer cells and tumor microenvironment, interfere with the establishment of stem cell niche in breast cancer, thereby providing therapeutic opportunities for targeted breast cancer treatment (35). RAB27A overexpression promotes the growth and metastasis of cancer in an exosome-dependent or independent manner. The correlation between the elevated levels of RAB27A and the presence of non-small cell lung cancer (NSCLC) and RAB27A might play a critical role in increasing chemosensitivity in NSCLC (36). Increased expression of RAB27B is linked to decreased survival rates among individuals with breast cancer, and this GTPase might impact the generation of angiogenic and prometabolic substances within the tumor microenvironment. In the tumor microenvironment, RAB27B may affect paracrine communication and play a significant role in cancer cell secretion (37). In addition, Rab27A and Rab27B are highly expressed in pancreatic cancer and are closely associated with tumor stage and vascular infiltration, and their mechanism of action may involve epithelial-mesenchymal transition (EMT) mediated by the MAPK/ERK signaling pathway, thereby promoting tumor invasion and metastasis (38, 39). The RAB35-regulated vesicle subpopulation facilitates communication between cancer cells and stromal cells, providing an environment conducive to tumor growth (40). RAB35 enhances the expression of integrin and promotes cell migration in cervical and breast cancer (41). The RAB37 protein acts as a tumor promoter in macrophages by mediating the exocytosis of IL-6 through the activation of PD-1 on CD8+ T cells, and its associated transport pathway can promote the formation of immunosuppressive TME (42).

RAB proteins transport a variety of cargoes and substrates to govern conventional and non-conventional vesicular secretion pathways thereby playing a role in remodeling TME. RAB GTPases are tightly controlled by cytokines in the microenvironment and enable aggressive cancer growth by delivering key factors to the tumor microenvironment. Therefore, there is a need to further understand how RAB regulates conventional vesicular transport during tumorigenesis and its role in tumor immunity and targeting RAB proteins could affect the relationship between cancer cells and the tumor microenvironment, thereby influencing tumor progression and providing therapeutic opportunities for targeted cancer therapies.

## 5 Regulation of autophagy by RAB proteins

As a highly conservative process of eukaryotic cellular recycling, autophagy breaks down cytoplasmic organelles, proteins, and macromolecules and circulates their breakdown products. Autophagy plays a key role in the development and progression of cancer (43), participates in the regulation of the immune microenvironment during the initiation and progression of tumors, and can promote tumor cell death, thereby playing a tumor suppressive role, and therefore can be used as a potential target to improve cancer treatment (44, 45). Dysregulated autophagy has an impact on health and disease.

There are several RAB proteins that are involved in autophagosome formation, including RAB1, RAB2, RAB5, RAB7, RAB9A, RAB11, RAB23, RAB32, RAB10, and RAB39A (**Table 1**, **Figure 2**). RAB1 produces dependent autophagy, which selectively inhibits this process through the GAS pathway (46). RAB1 has important regulatory functions in the growth, migration and survival of cancers. RAB1 affects neuroblastoma progression through interaction with the autophagy receptor protein optineurin (OPTN), regulation of autophagy-related signaling pathways, and influencing metabolic adaptations in tumor cells (47). In mammalian cells, Studies have shown that RAB2 is involved in regulating autophagosome formation. RAB2 is also linked to autophagy pathways and the Golgi apparatus through transport membranes and participates in different autophagy mechanisms. Study reports that RAB2 is required for autophagosome clearance in human breast cancer cells (48). RAB2 gene amplification occurs in a wide variety of human cancers and may contribute to tumorigenesis (49). RAB5 forms complexes that contribute to the early stages of autophagy (50). RAB5 can influence cancer cell progression by regulating autophagosome formation and maturation, influencing cancer cell adaptation to metabolic stress, and serving as a biomarker of antibody-drug coupling (ADC) efficacy. RAB11 is an endosomal recycling protein that contributes to the formation and maturation of autophagosome. Researchers reported that RAB11 loss can prevent autophagosomes from fusing with late endosomes, hinder autophagy, and eventually lead to apoptosis and promote tumorigenesis (51). Autophagosome formation and maturation can be regulated by RAB10. RAB10 can bind to the autophagy receptor OPTN, promoting mitochondrial autophagy (52). Down-regulation of miRNA targets RAB10 to activate the AMPK signaling pathway and inhibit proliferation, inducing autophagy and apoptosis in human hepatocellular carcinoma cells (53). Circular RNAs (circRNAs) regulates apoptosis and autophagy in gastric cancer by affecting microRNA expression of RAB10 (54). RAB39A regulates cancer stemness and tumorigenesis in sarcoma models (55), probably by regulating autophagy and lysosomal fusion. RAB7, RAB8B and RAB24 play a crucial role in the maturation of autophagosome. By regulating lysosomal fusion, the RAB7 effector can promote the degradation of neuronal autophagy lysosome (56). RAB7 induces mitophagy and plays a pivotal tumor-suppressing role in gastric cancer (57). Reducing the expression of RAB7 inhibited the autophagy level of gastric cancer cells, suppressed the inhibitory effect in the proliferation, migration and invasion of gastric cancer cells, and promoted cell apoptosis (58). RAB23 is localized to autophagosomes and inhibits cell growth and proliferation and induces apoptosis in breast cancer cells (59). Furthermore, RAB22A was shown to facilitate anti-tumor immunity by mediating the formation of non-typical autophagosomes (60). Depletion of RAB escort protein 1 (REP1) blocks autophagosome formation, increases megacellular drinking, and modulates intracellular nutrient levels and mTOR activity. These suggest that REP1-mediated intracellular metabolism and degradation processes are key regulators of cancer cell survival and are expected to be potential targets for cancer therapy (61).

As key regulators of vesicular transport and molecular “switches” for vesicle trafficking, many RAB GTPases play a critical role in the autophagy process. Autophagy, a cellular degradation and recycling mechanism, has a dual role in cancer biology. On one hand, it acts as a protective mechanism by preventing early tumor development; on the other hand, it supports metabolic adaptation, maintenance, and survival of established and metastatic tumors (62). This dual nature of autophagy, both promoting and inhibiting cancer, highlights its significant contribution to tumor initiation and progression. Furthermore, autophagy is involved in shaping an immunosuppressive tumor microenvironment, making it a potential therapeutic target for cancer treatment. RAB proteins, through their regulation of autophagy and related vesicular transport processes, are emerging as crucial players in cancer development. For instance, specific RAB proteins modulate autophagosome formation, maturation, and fusion with lysosomes, thereby influencing autophagic flux. Dysregulation of these RAB-mediated processes can lead to either excessive or insufficient autophagy, both of which can drive tumor progression.

## 6 Anticancer drugs are resistant to RABs

Drug resistance refers to the tolerance of microorganisms, parasites and tumor cells to the effects of chemotherapy drugs. Once drug resistance occurs, the chemotherapy effects of drugs will decrease significantly. At present, tumor drug resistance is the main problem that limits the efficacy of tumor chemotherapy drugs. Cancer cells are also resistant to immunotherapeutic drugs, which may lead to the rapid recurrence of cancer/disease progression and ultimately cause the death of patients.

For example, the miRNA expression can increase cisplatin resistance in lung cancer stem cells by inhibiting RAB6 (63). RAB6B and RAB6C are associated with drug sensitivity. The knockdown of RAB6B inhibits liver cancer cell proliferation and promotes apoptosis, thus improving drug sensitivity. Similarly, the overexpression of RAB6c induces the intracellular accumulation of several anticancer drugs. This may contribute to the development of drug resistance in breast cancer cells (64). Moreover, RAB7 can mediate resistance to cisplatin chemotherapy in extracellular vesicles, and RAB7 expression is downregulated in many cisplatin-resistant cell lines compared to sensitive cell lines (65). RAB7 affects drug chemotherapy resistance in cervical cancer cells by regulating advanced endocytosis pathways and extracellular vesicle secretion (17). RAB27A has also been related to resistance to some conventional chemotherapy drugs. A downregulation of RAB27A can increase chemotherapy sensitivity in lung cancer cells (36). Rab27B enhances chemoresistance of tumor cells by promoting exosome release in hepatocellular carcinoma (66). In addition, secretory RAB8 is another RAB involved in chemotherapeutic drug resistance. Cells resistant to platinum compounds generally express high levels of RAB8, and overexpression of RAB8 can enhance cisplatin resistance by increasing its secretion of cisplatin-resistant proteins, a potential target for cancer chemotherapy. RAB-like protein 1 A (RBEL1A) is up-regulated in breast tumor cells, promotes cell proliferation, cell cycle and invasion, induces cisplatin sensitivity, and RBEL1A induction after cisplatin treatment inhibits chemosensitivity (67).

Cancer cells undergo dramatic metabolic adaptations to meet increased bioenergetic demands, leading to resistance to treatment. Tumor cells can influence the proliferative, migratory, and apoptotic stages of a tumor by developing drug resistance. Several RAB proteins have been associated with resistance to anticancer drugs, and the cytosolic action of RAB proteins, whose dysregulation affects tumor cell resistance and alterations in the tumor microenvironment in tumorigenesis. Targeting RAB proteins further provides an opportunity for therapeutic strategies to reduce cancer progression and anticancer resistance, providing new targets for cancer therapy.

## 7 The role of RAB proteins in metabolism

It has been shown that transport and metabolic signaling pathways intersect, meaning that vesicular transport can influence the regulation of metabolic signaling (68). RAB GTPases play an essential role in the regulation of vesicular transport and also influence and participate in cellular metabolic processes. RAB GTPases can regulate the transport of GLUT (glucose transporter) and the formation of lipid droplets (LD) as well as the processes of glucose metabolism and lipid metabolism in cancer cells.

For muscle contractions to occur, glucose is an important fuel, and glucose metabolism plays an important role in health. The glucose transporter (GLUT) is necessary for muscle and fat to utilize dietary glucose. We know that glucose transporter (GLUT), a transmembrane protein family that affects the cell’s ability to absorb extracellular glucose, is involved in glucose metabolism, inflammatory reaction, and immune response.

The primary glucose transporter GLUT4 transports glucose from intracellular vesicles to the cytoplasm (69). The RAB protein plays an important role in metabolic reprogramming, and studies have demonstrated that GLUT4 transport is mediated by RAB proteins. A RAB-GTPase is a critical regulator of GLUT4 transport, coordinating its translocation to the plasma membrane (70). Some RAB proteins participate in GLUT4 transport, such as RAB8A, RAB10, RAB20, RAB13 (**Table 1**, **Figure 2**). Overexpression of RAB8A inhibits insulin-induced GLUT4 transport processes in skelet al muscle cells by regulating exocytosis of GLUT4 vesicles (69). Reduction of RAB8A protein inhibits breast cancer proliferation, migration, and invasion, thereby decreasing the survival of cancer patients (71). The RAB10 GTPase plays an important role in glucose metabolism in cells, mainly related to GLUT4 exocytosis in fat cells (72). An insufficient expression of RAB20 in hepatoma cells leads to the release of EVs, and a decrease in triosephosphate isomerase 1 (TPI1) levels promotes aerobic glycolysis and promotes the development of liver cancer (73). Glucose transport and autophagy are important functions of RAB21, a small GTPase. RAB21 depletion affects the glucose uptake process, thereby increasing autophagy flux (74), while also inhibiting tumor progression *in vivo*, indicating RAB21 plays a role in carcinogenicity. In addition, RAB-GAP can act on RAB35 to regulate GLUT4 transport in insulin-stimulated adipocytes (75) and it plays a role in cancer invasion, metastasis and immune escape. A study showed that RAB7 depletion reduced mTOR overactivation, decreased glucose consumption and ROS overproduction, and increased the number of healthy mitochondria (76). Gamma-glutamyl transferase 7 suppresses gastric cancer by cooperating with RAB7 to induce mitophagy (57). Therefore, we hypothesized that RAB7 may play an important role in cancer progression through cellular metabolism. RAB32 supports mTORC1 signaling to regulate proliferation and metabolism in hepatocellular carcinoma by binding to lysosomes (68).

The RAB proteins are involved in GLUT4 transport, which controls glucose metabolism, allowing tumor cells to grow and proliferate (77). GLUT4 acts as an insulin-responsive glucose transporter from GLUT4 storage vesicles to the cell membrane. So, we speculate that GLUT4 transport is mediated by RAB proteins, affecting the development of glucose metabolism in tumors. We can target glycolytic pathways by modulating RAB proteins as a therapeutic approach to inhibit cancer progression. RAB protein is involved in tumor cell autophagy, tumor microenvironment, and glucose metabolism, and these processes are involved in the tumors (68).

## 8 Conclusion and future perspective

**Figure 3:**
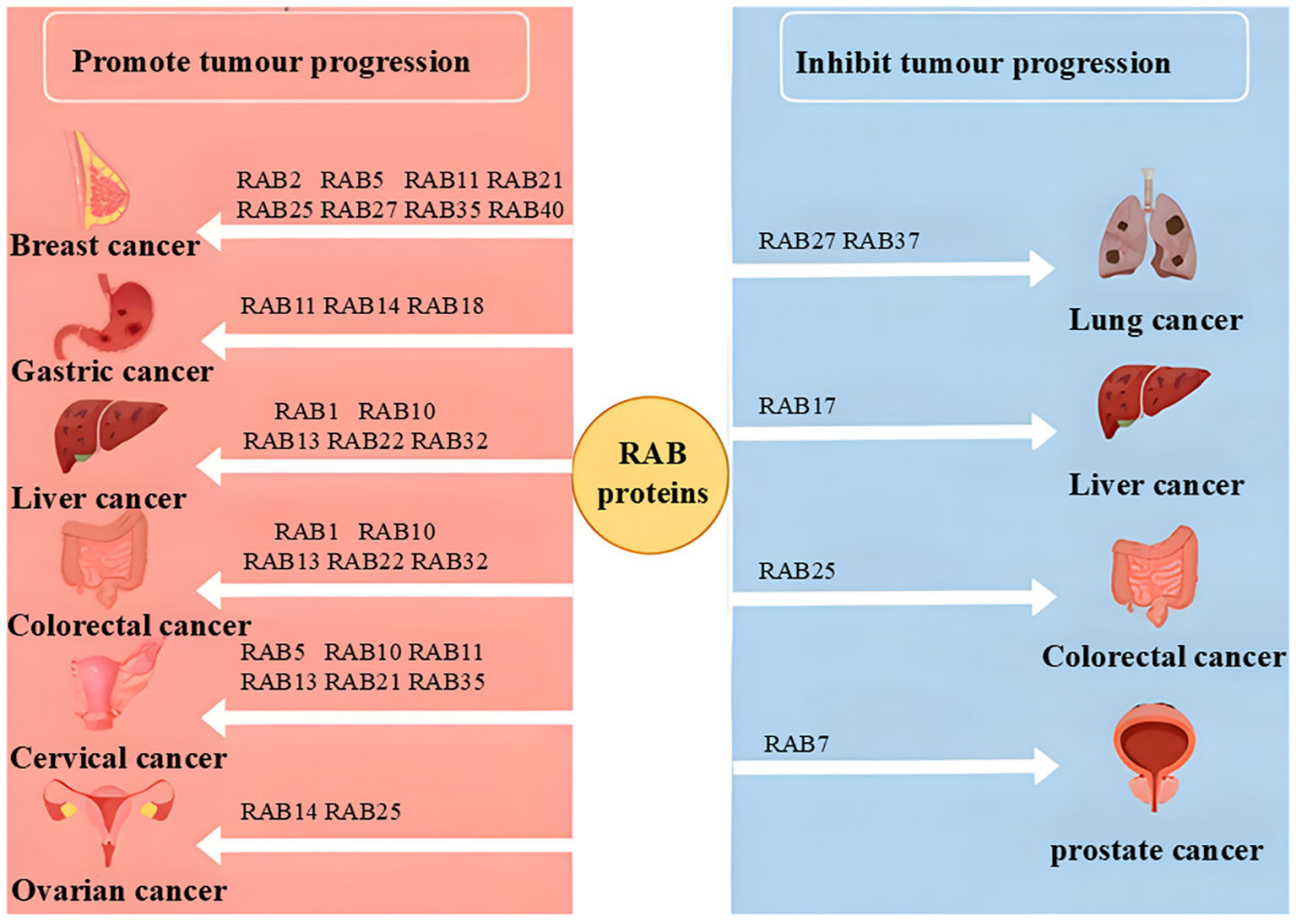
Schematic representation of oncogenic and suppressor RAB proteins in different cancers.

The RAB family, the largest group within the RAS superfamily, plays a pivotal role in cancer progression by regulating vesicular trafficking, tumor microenvironment, autophagy, and glucose metabolism, thereby influencing metastasis, proliferation, and cell cycle control (**Figures 2**, **3**). Unlike other RAS superfamily members, such as RAS, RHO, and ARF, which primarily mediate signal transduction and cytoskeletal dynamics, RAB proteins exert their unique functions through the modulation of vesicle transport. For instance, while RAS mutations often drive uncontrolled cell proliferation via pathways like MAPK signaling, RAB proteins impact tumor development by altering vesicular trafficking and secretion, which in turn affects the tumor microenvironment, cellular metabolism, and metastatic processes.

In recent years, the role of the RAB family in cancer has garnered increasing attention, with its critical function in membrane trafficking offering unique therapeutic targets, particularly in strategies aimed at altering cancer cell behavior by modulating intracellular transport. RAB proteins play a pivotal role in the tumor microenvironment by regulating vesicular trafficking and exosome secretion. Exosomes, containing DNA, RNA, proteins, lipids, and lipoproteins, are essential for intercellular communication, influencing gene expression and functionality in recipient cells. For instance, mRNA and miRNA can serve as tumor-specific biomarkers for diagnosis, while modulating the activity of specific RNAs may offer novel therapeutic approaches. Exosomal proteins, such as growth factors, cytokines, and enzymes, may promote tumor angiogenesis or immune evasion. Targeting these RNAs and proteins to develop specific diagnostic markers or small-molecule inhibitors could enhance early tumor detection, improve treatment precision, and provide deeper insights into RAB-mediated tumor progression. Furthermore, integrating multi-omics technologies to comprehensively analyze these components will help unravel the complex roles of RAB proteins in the tumor microenvironment.

RAB proteins are essential for normal cellular physiology, but their dysregulation in cancer can lead to detrimental outcomes, closely linked to tumor initiation, progression, and metastasis. Depending on the cellular context, RAB proteins can function as either oncogenes or tumor suppressors (**Figure 3**). This dual role makes the RAB family particularly intriguing for understanding the complex mechanisms of cancer progression, though it also complicates therapeutic strategies. For instance, RAB3B has been identified as a hub gene for diagnosis and prognosis prediction in various cancers, including lung adenocarcinoma, glioma, and colorectal cancer. Additionally, RAB22A has been shown to promote anti-tumor immunity by mediating the formation of atypical autophagosomes (60).

The functional diversity of RAB proteins across different cancers is closely tied to their intracellular mechanisms. For instance, RAB27A and RAB27B exhibit distinct roles in various cancers: in liver cancer, RAB27B enhances chemotherapy resistance by promoting exosome release, while in pancreatic cancer, their overexpression correlates with tumor stage and vascular invasion, demonstrating oncogenic properties. However, in certain digestive system tumors, they may act as tumor suppressors (39, 66). These functional differences may arise from variations in expression levels, interactions with other signaling pathways, and differences in the cellular microenvironment. Tissue specificity and cancer subtypes also influence RAB protein functions. For example, RAB25 acts as an oncogene in ovarian, breast, and gastric cancers but suppresses tumor growth in colon and head-neck cancers, likely due to its subcellular localization and roles in different tissues (78). Additionally, some RAB proteins exhibit dual functions depending on the cellular context and dynamic changes during tumor progression. For instance, RAB27A not only promotes metastasis in pancreatic cancer but also modulates the tumor immune microenvironment by regulating exosome secretion in immune cells (38). These complexities underscore the necessity of in-depth research into the mechanism-dependent roles of RAB proteins in cancer progression.

Despite the significant potential of the RAB family in cancer therapy, targeting RAB proteins presents unique challenges. Unlike the RAS family, which primarily targets mutant proteins, RAB proteins share high sequence and structural similarity, exhibit functional complexity, and lack distinct binding sites, making specific targeting difficult. Additionally, RAB protein activity is regulated by multiple upstream regulators and downstream effectors, meaning targeting a single RAB protein may yield limited therapeutic effects. Currently, the absence of well-defined small-molecule inhibitors for RAB proteins further restricts the development of targeted therapies. However, the critical role of RAB proteins in vesicular trafficking offers a promising avenue for cancer treatment, such as improving drug delivery or enhancing immune cell function by modulating vesicle transport.

Given the complexity of RAB proteins and the challenges in targeting them directly, focusing on their effector proteins or downstream signaling pathways may prove more effective. RAB proteins exert their functions through interactions with effectors, such as SAND-1, which mediates vesicle transport from late endosomes to lysosomes in the case of RAB7 (79). Moreover, downstream pathways of RAB proteins play significant roles in cancer; for example, RAB34 regulates tumor cell adhesion and migration by interacting with integrin β3 (30). Targeting these effectors or pathways could overcome the limitations of single RAB protein targeting, though their dual functionality in upstream and downstream contexts necessitates personalized treatment strategies tailored to specific tissues and cellular backgrounds. Combining RAB-targeted therapies with existing treatments, such as immunotherapy or chemotherapy, through synergistic approaches may enhance therapeutic outcomes. Additionally, leveraging CRISPR/Cas9 or RNA interference to selectively knock down oncogenic RAB proteins or their regulators, or developing highly selective small-molecule inhibitors, could be viable strategies, particularly in cancers with specific RAB protein overexpression. While targeting RAB proteins represents a novel therapeutic approach, their potential dual roles require careful evaluation. Addressing these challenges could provide valuable insights for cancer research and treatment.
